# The Physicians of Dantzic Sixty Years Since

**Published:** 1843-06

**Authors:** 


*The Physicians of Dantzic Sixty Years Since.*—These reverend gentlemen enjoyed the title of excellency, and not only in their own houses and from their own servants, but in society generally; only very intimate friends could sometimes venture on a respectful "Herr Doctor." Their head was covered with a snow-white, powdered, full-bottomed periwig, with three tails, one of which hung down the back, while the others floated on the shoulders. A scarlet coat embroidered with gold, very broad lace ruffles and frill, white or black silk stockings, knee and shoe buckles of sparkling stones, or silver gilt, and a little, flat, three cocked hat under the arm, completed the toilette of these excellencies. Add to this a pretty large cane, with a gold head, or mermaid carved in ivory, upon which, in difficult cases, to rest the chin—and certainly every one will admit the impossibility of so much as thinking of an innovation in their presence.—*Lond. Lancet,* from *Edin. Review*, Feb. 1842.

